# Misfolding of transthyretin in vivo is controlled by the redox environment and macromolecular crowding

**DOI:** 10.1016/j.jbc.2024.108031

**Published:** 2024-11-28

**Authors:** Sanduni Wasana Jayaweera, Melisnur Sahin, Fabian Lundkvist, Alice Leven, Laura Tereenstra, Joel Bäckman, Anushree Bachhar, Fouzia Bano, Intissar Anan, Anders Olofsson

**Affiliations:** 1 Department of Clinical Microbiology, Umeå University, Umeå, Sweden; 2 Department of Public Health and Clinical Medicine, Umeå University, Umeå, Sweden

**Keywords:** transthyretin, redox, macromolecular crowding, amyloid, cysteine, disulfide

## Abstract

Transthyretin (TTR) amyloidosis is a progressive disorder characterized by peripheral neuropathy, autonomic dysfunction, and cardiomyopathy. The precise mechanism by which TTR misfolds and forms fibrils *in vivo* remains incompletely understood, posing challenges to the development of effective therapeutics. In this study, we reveal that the recently identified nonnative pathological species of TTR (NNTTR), which is enriched in the plasma of *ttr-val30met* gene carriers, exhibits strong amyloidogenic properties, making it a promising therapeutic target. Notably, we demonstrate that NNTTR formation is dependent on an intermolecular disulfide bond and can be promoted by oxidative conditions while being effectively suppressed by reducing agents. The formation of this disulfide bond is incompatible with the native TTR fold, thereby necessitating structural flexibility. We further show that this required flexibility can be constrained using tetramer-stabilizing drugs, thereby suppressing NNTTR formation. Interestingly, the flexibility is also hindered by macromolecular crowding, and NNTTR formation is strongly suppressed by the high protein concentration in plasma. This suppression is released upon dilution, which thus promotes NNTTR formation in areas with lower protein content, highlighting a potential link to the interstitial space, brain, and vitreous body of the eye, where TTR-amyloid is frequently observed. In summary, we demonstrate that NNTTR displays strong amyloidogenic features, underscoring its potential as a therapeutic target. We identify the redox environment and macromolecular crowding as key modulatory factors. Our findings propose a mechanistic explanation for TTR misfolding and suggest a novel therapeutic approach.

Transthyretin (TTR)-amyloidosis is a progressive systemic human disorder characterized by the accumulation of amyloid fibrils of the extracellular protein TTR in various tissues (1). The disease can be both inherited and acquired, with the inherited form (ATTRv amyloidosis) linked to mutations in the TTR gene, and the acquired form (ATTR wt amyloidosis) associated with amyloid derived from the native protein sequence. ATTRv amyloid deposits are frequently found along peripheral nerves, in the kidneys, intestines, and the heart, causing hypertrophic cardiomyopathy, sensory and motoric dysfunction, and gastrointestinal problems (2, 3). More than 130 mutations have been linked to ATTRv amyloidosis where a mutation resulting in an exchange of valine to methionine in position 30 (*ttr-val30met*), represents one of the most common and well-studied variants (4). The onset of ATTRv amyloidosis usually occurs in midlife (5, 6), while ATTR WT amyloidosis, previously referred to as senile systemic amyloidosis, has a later onset (7). Notably, ATTR WT deposits are found in approximately 25% of all men around 80 years of age (8).

TTR is an extracellular 127-residue homotetrameric protein mainly produced by the liver, but expression also occurs in the choroid plexus of the brain, the retina of the eye, and the pancreatic islets of Langerhans (9). The protein is involved in the transport of the thyroxine T4 hormone, and indirectly also in the transport of retinol, where it binds the holo-retinol-binding protein (10).

The conversion of native TTR into an amyloid fold requires partial denaturation and dissociation of its tetrameric fold (11, 12, 13). Amyloidogenic mutations in TTR are associated with lower stability of the protein, thus enhancing this process (14). A reduction in the rate of tetramer dissociation can, however, be obtained *via* ligand stabilization using the binding sites for the thyroxine T4 hormone (12). This approach, which today is in clinical use with the drugs diflunisal and tafamidis, stabilizes the native state and thus lowers the rate of amyloid formation, (15, 16). TTR-amyloidosis is today also treated by downregulating the liver-specific expression of TTR, using RNA interference and antisense oligonucleotides (17, 18).

The mechanism by which the TTR misfolds and forms amyloid *in vivo* has so far not been fully understood which has hampered the development of new therapeutics. In a recent study based on a sandwich ELISA approach, circulating nonnative variants of TTR (NNTTR), were identified in human plasma from *ttr-val30met* carriers (19). Low levels of NNTTR notably also correlated with a better prognosis, supporting their link to the pathology (19). Using the monoclonal antibody MAB_39-44_, known to bind a cryptic epitope on TTR, we can here corroborate their finding and verify the presence of NNTTR in plasma from *ttr-val30met* carriers. Through the characterization of plasma-derived NNTTR from *ttr-val30met* carriers, we interestingly discovered that their formation is dependent on a nonnative intermolecular disulfide linkage between subunits of the tetramer.

TTR contains a single cysteine, located at position 10, and an intermolecular disulfide bond between subunits is notably a well-known characteristic feature of TTR-amyloid *in vivo* (20, 21). An intermolecular disulfide bridge with a preserved tetrameric assembly is however not compatible with a preserved native fold. The new state which consequently is locked in an alternative fold, interestingly displays a highly increased sensitivity toward proteolytic digest. Proteolytic fragmentation is a common feature of TTR-amyloid *in vivo* (21) and also a well-known means to increase the rate of amyloid formation, both *in vitro* and *in vivo* (22, 23, 24, 25). In conclusion, the observed amyloidogenic features of NNTTR highlight it as a relevant therapeutic target for intervention, and factors with the ability to modulate NNTTR are thus of interest to identify.

We can here expose the redox environment as a strong modulator of NNTTR and while oxidizing conditions can promote the formation, NNTTR can be effectively suppressed by reducing agents. Concerning the oxidation of TTR, it can be mediated through different routes and *in vitro*, the addition of the oxidating agent diamide effectively induces a disulfide bond between subunits of the tetramer. However, in plasma, the single cysteine of TTR is to a high extent already oxidized by various disulfide-linked adducts including cysteinylation, cysteine-glycinylation, and glutathionylation, and frequently only around 10 to 30% of the cysteines on TTR reside in their reduced thiol state (26, 27, 28, 29, 30). Although disulfides are strong covalent bonds, they are also dynamic due to their susceptibility to react with thiols. The reaction is known as “disulfide exchange” or “disulfide shuffling” and the net result is an interchange of the substituents (31). The ability to form a disulfide bond between two monomers of the tetramer is thus controlled by the relative proportion of bound *versus* free thiols. A stoichiometric excess of free thiols can thus lower the proportion of reactions between cysteines within the tetramer and we can here expose strong suppressing effects from the reducing agents β-mercaptoethanol (BME), GSH, tris(2-carboxyethyl) phosphine (TCEP), and N-acetylcysteine (NAC). Interestingly, NAC is a well-known drug and food additive, with a broad safety profile, and its potential therapeutic use is further discussed below.

Due to the distance between the cysteine residues in the native tetramer, the conversion from a native fold to a disulfide-linked tetramer requires a certain degree of flexibility. The less stable structure of TTR-V30M makes it more prone to forming NNTTR compared to WT TTR, which likely explains why NNTTR is enriched in the plasma of TTR-Val30Met carriers. However, protein flexibility can be restrained by external factors, and we can here, interestingly, show that the formation of NNTTR in plasma is strongly suppressed by the macromolecular crowding effect caused by high protein concentrations. When plasma is diluted, this suppressive effect is diminished, resulting in a strong promotion of NNTTR formation.

Interestingly, the suppressive role of macromolecular crowding offers a potential link to environments where TTR amyloid is found *in vivo*, such as the interstitial space and the vitreous body of the eye, where protein concentrations are significantly lower. Consistent with this rationale, we also demonstrate that tetramer-stabilizing ligands effectively prevent NNTTR formation, further supporting this hypothesis.

In summary, this work uncovers two new areas in TTR amyloid research—highlighting the influence of the redox environment and macromolecular crowding—and provides a mechanistic explanation for TTR misfolding *in vivo*. These findings suggest potential new strategies to counteract misfolding, with both therapeutic and preventive implications.

## Results

### MAB_39-44_ effectively detects NNTTR in human plasma from ttr-val30met carriers

**Figure 1:**
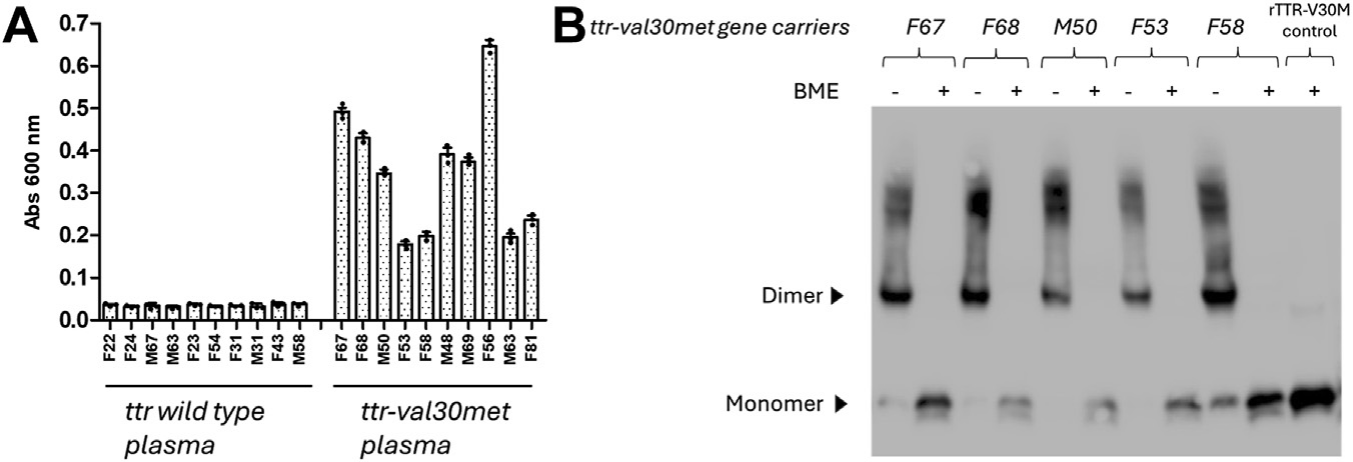
**NNTTR from *ttr-val30met* carriers contains a high proportion of a nonnative disulfide bond.***A*, analysis of the levels of NNTTR in plasma from ten heterozygote carriers of *ttr-val30met* and 10 controls with the *ttr wt* gene, conducted using a sandwich-based ELISA with MAB_39-44_. All plasmas were diluted 400X before analysis. The bars show sex (female (F) and male (M)) and age, as well as standard deviation. *B*, SDS-PAGE and Western blot analysis after immunoprecipitation of plasma from five *ttr-val30met* carriers. The immunoprecipitated material was analyzed after boiling in SDS-loading buffer under nonreducing *versus* reducing conditions, followed by SDS-PAGE and Western blotting. Detection of TTR was performed using the polyclonal rabbit anti-TTR antibody TTR_49-127_ according to the standard procedures. The sex (female (F) and male (M)) and age of the individuals are indicated in the figure. rTTR-V30M boiled in SDS-loading buffer under reducing conditions was included as a control. NNTTR, nonnative variants of TTR; rTTR, recombinant TTR; TTR, transthyretin.

Using an ELISA-based setup, the presence of NNTTR in plasma from *ttr-val30met* carriers was recently discovered (19). The mouse monoclonal anti-TTR antibody, MAB_39-44_ has been shown to detect a cryptic epitope (32) and through a sandwich-based ELISA setup, we could confirm a high selectivity for NNTTR in favor of native TTR, also regarding this antibody. The presence of NNTTR in plasma could thus be corroborated, and Figure 1*A* shows the responses of 10 carriers of *ttr-val30met* and 10 *ttr wt* controls, including age and gender. To further characterize the NNTTR, we performed immunoprecipitation using MAB_39-44_ on plasma from *ttr-val30met* carriers followed by SDS-PAGE and Western blotting, under nonreducing *versus* reducing conditions. The results interestingly showed that the TTR captured by the MAB_39-44_ antibody was greatly enriched by a disulfide-linked dimeric form. Figure 1*B* shows a representative result from five *ttr-val30met* carriers as well as a recombinant TTR-V30M (rTTR-V30M) control.

The immunoprecipitation showed that the NNTTR present in plasma from the *ttr-val30met* carriers contained an intermolecular disulfide bond, seen as a dimeric band on the Western blot, which could be effectively dissociated into monomers using BME in the sample buffer. The immunoprecipitated material also contained a small fraction of higher molecular weight assemblies. Notably, also these assemblies are effectively dissociated into monomers in the presence of a reducing agent, indicating that their formation relies on disulfide bonds. From a technical standpoint, it is important to note that protein transfer rates from SDS-PAGE to the membrane vary with molecular size. To better highlight the presence of dimers and higher molecular weight assemblies, the electrophoretic transfer time was extended, which caused some of the monomers to pass through the membrane, resulting in a slightly weaker monomeric band than expected.

### The formation of NNTTR correlates with the formation of a disulfide bond between subunits

**Figure 2:**
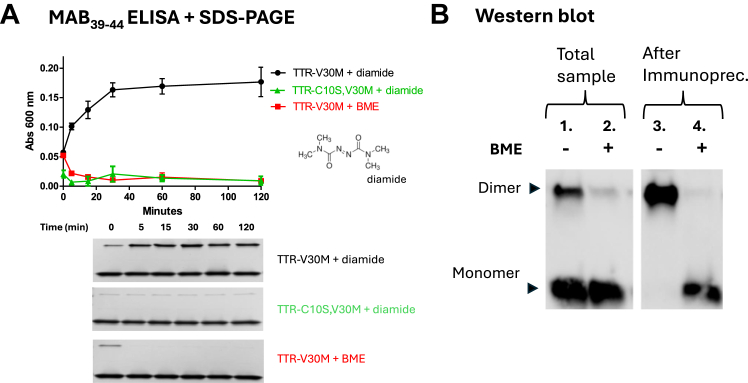
**Disulfide formation between subunits of TTR correlates with formation of NNTTR.***A*, the presence of NNTTR was assessed using the MAB_39-44_ sandwich-based ELISA on rTTR-V30M (4 μM in PBS), incubated at 37 °C for 0 to 2 h with either 40 μM diamide or 1 mM BME. To exclude potential nonspecific effects from diamide, the rTTR-C10S, V30M mutant, lacking cysteines, was incubated with 40 μM diamide under identical conditions. All proteins were monitored using nonreducing SDS-PAGE stained for proteins. *B*, SDS-PAGE and Western blot analysis were performed on rTTR-V30M, incubated overnight at 37 °C in PBS with 40 μM diamide. Lanes 1 and 2 show the total sample, before immunoprecipitation, boiled in nonreducing *versus* reducing SDS-loading, respectively. Lanes 3 and 4 show the analysis of the material captured by immunoprecipitation with MAB_39-44_. TTR was detected by Western blotting using the polyclonal rabbit anti-TTR_49-127_ antibody, following standard procedures. BME, β-mercaptoethanol; NNTTR, nonnative variants of TTR; rTTR, recombinant TTR; TTR, transthyretin.

The finding that NNTTR in human plasma contained a high proportion of a disulfide-linked dimer prompted us to investigate whether NNTTR formation could also be induced by promoting the formation of a disulfide bond. Figure 2*A* shows a time-dependent study of recombinant rTTR-V30M as a function of 40 μM of diamide, a commonly used oxidizing agent that promotes the formation of disulfide bonds (the structure of diamide is included in Fig. 2*A*). The formation of NNTTR was monitored using the MAB_39-44_ sandwich-based ELISA, and the results demonstrate that enhancing intermolecular disulfide bond formation significantly promotes NNTTR formation. To confirm the correlation between disulfide-linked dimer formation and MAB_39-44_ reactivity, and to rule out any secondary effects from diamide, two controls were included in the analysis: rTTR-V30M incubated with BME, and a mutant lacking the single cysteine (rTTR-C10S, V30M) incubated with 40 μM diamide. Additionally, all time points were also analyzed using SDS-PAGE stained for protein. The results confirm that NNTTR formation is directly linked to a disulfide bond formation between subunits, with no secondary effects from diamide.

To further investigate the properties and selectivity of MAB_39-44_ immunoprecipitation on rTTR-V30M was performed using the same procedure as described above. The results revealed that MAB_39-44_ essentially exclusively binds to the disulfide-linked form of rTTR-V30M, showing little to no affinity for its native, nondisulfide-linked counterpart, Figure 2*B*.

### Disulfide-linkage of TTR-V30M does not impair fibril formation

**Figure 3:**
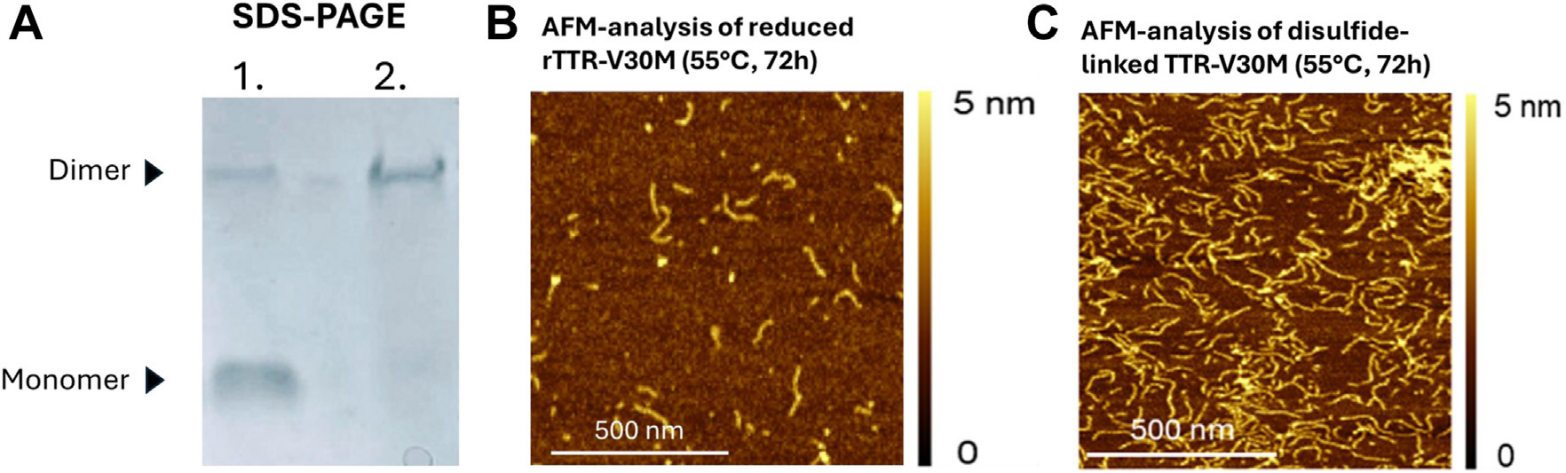
**Isolation of disulfide-linked TTR-V30M and AFM analysis.***A*, SDS-PAGE stained for protein using Coomassie brilliant blue, before and after isolation of disulfide-linked rTTR-V30M using anion-exchange chromatography. *B*, AFM analysis of fully reduced TTR-V30M after incubation in PBS, pH 7.4, at 55 °C for 72 h (scale bar represents 500 nm). *C*, AFM analysis of disulfide-linked TTR-V30M after incubation in PBS, pH 7.4, at 55 °C for 72 h (scale bar represents 500 nm). AFM, atomic force microscopy; rTTR, recombinant TTR; TTR, transthyretin.

Disulfide-linked TTR is abundantly found in *ex vivo* isolated amyloid (21, 33), but it is not elucidated if this bond forms after fibril formation or if two subunits linked by a disulfide bond at position 10 can serve as a precursor for amyloid formation. To address this, the disulfide-linked fraction of rTTR-V30M was purified using anion-exchange chromatography (Mono-Q 5/5, Cytiva) in urea, followed by buffer exchange to PBS. Figure 3*A* presents an SDS-PAGE analysis of the starting material after diamide treatment, along with the enriched fraction of disulfide-linked rTTR-V30M following separation by anion-exchange chromatography. The conversion of native TTR into amyloid fibrils under physiological pH has previously been shown to be enhanced by a temperature of around 55 °C degrees and a full conversion into fibrils requires approximately 72 h (34, 35). This approach was therefore used on both reduced rTTR-V30M and the isolated disulfide-linked rTTR-V30M. Morphology of the formed aggregates was followed by atomic force microscopy (AFM) and the result showed that both the reduced rTTR-V30M (Fig. 3*B*) and the disulfide-linked TTR-V30M (Fig. 3*C*) acquired a fibrillar morphology.

### The disulfide-linked TTR retains a tetrameric assembly but acquires a high sensitivity for proteolytic digest

**Figure 4:**
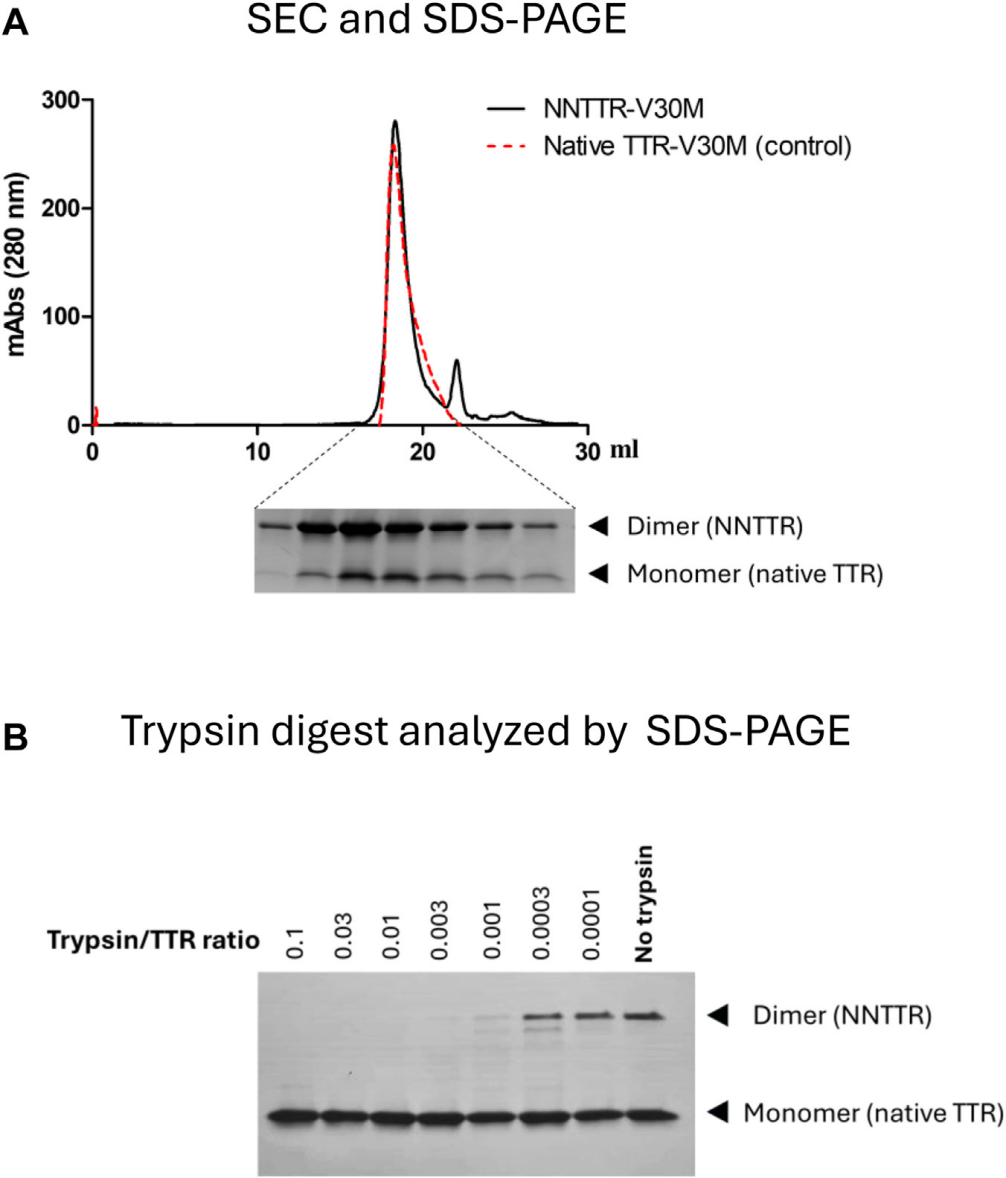
**The disulfide-linked NNTTR retains a tetrameric structure but acquires a high sensitivity for proteolytic digestion by trypsin.***A*, SEC analysis of rTTR-V30M having a fraction of NNTTR corresponding to around 70%, (*black line*), and fully reduced native rTTR-V30M (*red line*). The inset shows the fractions of the NNTTR after SEC analyzed on SDS-PAGE verifying the presence of a high fraction of disulfide-linked dimers. *B*, nonreducing SDS-PAGE analysis of rTTR-V30M containing 25% of NNTTR and 75% native tetramers after exposure to various concentrations of bovine trypsin in PBS for 1 h at 37 °C. NNTTR, nonnative variants of TTR; SEC, size-exclusion chromatography; rTTR, recombinant TTR; TTR, transthyretin.

To further characterize the structural integrity of NNTTR it was subjected to size-exclusion chromatography (SEC). As shown above, the conversion into the disulfide-linkage can be performed in PBS by the addition of diamide which frequently mediates around 25% conversion of the total sample. However, by including urea in the mixture of TTR during incubation with diamide, the disulfide-linked fraction can be further enhanced. To accurately determine the exclusion volume of the disulfide-linked species the rTTR-V30M was therefore prepared using a combination of diamide and 6M urea which converted around 70 to 80% to its disulfide-linked form. Before analysis, both urea and diamide were removed through a buffer exchange step using a PD 25 column (Bio-Rad) equilibrated with PBS. The exclusion volume of rTTR-V30M enriched in the disulfide-linked form was subsequently analyzed using a 10/300 Superose 6 column, (Cytiva), equilibrated in PBS, Figure 4*A*. The result displays that the dimer-enriched rTTR-V30M elutes with an exclusion volume corresponding to a native tetramer. A high proportion of disulfide-linked subunits was verified through SDS-PAGE analysis over the elution profile. The elution profile of a fully reduced native sample of rTTR-V30M included as a control shows an essentially complete overlap, Figure 4*A* suggesting that the constraints caused by the intermolecular disulfide bond do not prevent a tetrameric assembly of NNTTR.

To further probe the structural properties of NNTTR *versus* native TTR it was subjected to proteolytic digest. Figure 4*B* shows a nonreducing SDS-PAGE analysis of an rTTR-V30M sample containing around 25% NNTTR (seen as dimers on SDS-PAGE) and 75% native rTTR-V30M (seen as monomers on SDS-PAGE), exposed to different concentrations of bovine trypsin. Through this approach, the native TTR as well as the NNTTR is exposed to identical concentrations of the protease at each point of measurement. The results show that the NNTTR form is between 80 to 240 times more sensitive to proteolytic digest as compared to the native, nondisulfide-linked form, which is essentially unaffected.

### NNTTR formation can be controlled by the redox environment and is effectively suppressed by reducing agents

**Figure 5:**
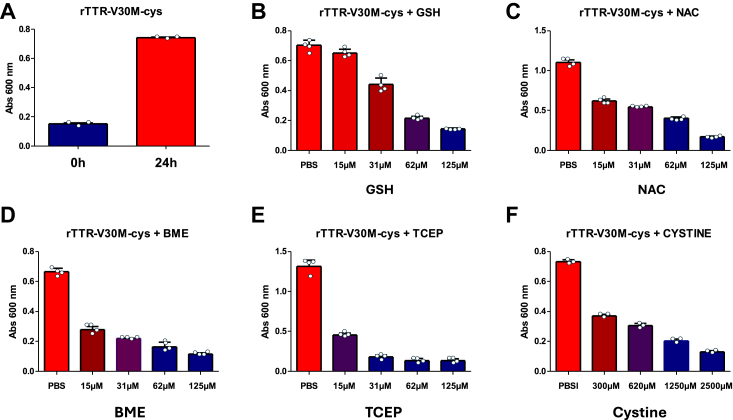
**The formation of NNTTR is effectively suppressed by reducing agents.** The relative formation of NNTTR from rTTR-V30M-Cys at 4 μM incubated in PBS at 37 °C for 24 h, using the MAB_39-44_ sandwich-based ELISA. *A*, shows the relative increase of NNTTR between 0 and 24 h. *B*–*F*, show the formation of NNTTR after 24 h as a function of 0 to 125 μM GSH; 0 to 125 μM NAC; 0 to 125 μM BME; 0 to 125 μM TCEP; and 0 to 2500 μM cystine, respectively. All samples were diluted 5000X in PBS-T before analysis and error bars show the standard deviation. All experiments have been repeated at least three times. BME, β-mercaptoethanol; NAC, N-acetylcysteine; NNTTR, nonnative variants of TTR; PBS-T, PBS supplied with 0.3% Tween-20; rTTR, recombinant TTR; TCEP, tris(2-carboxyethyl) phosphine.

The above results show that the oxidizing reaction that forms a disulfide bond between subunits of TTR correlates with the formation of NNTTR. The cysteine 10 of TTR *in vivo* is, however, highly modified by disulfide-linked adducts, and frequently only 10 to 30% of the TTR resides in a reduced thiol state (26, 27, 28, 29, 30, 36, 37). As mentioned earlier, thiols can react with disulfides. By incubating rTTR-V30M with an excess of cystine, the disulfide-linked form of cysteine, a cysteine adduct can effectively be conjugated to the protein. Mass spectrometry analysis showed that around 90% of the TTR in this investigation became cysteinylated. Maintaining an external stoichiometric excess of cystine effectively prevents the formation of intermolecular disulfide bonds between TTR subunits, as the continuous reaction with free cystines in solution is more likely than the formation of a disulfide bond within the TTR tetramer. However, after removing excess free cystine *via* SEC, a new equilibrium is established. This approach exposes a potential route of NNTTR formation *in vivo* as well as a controlled way to monitor the conversion of native TTR into NNTTR. Figure 5*A* illustrates the time-dependent conversion of cysteinylated recombinant TTR (rTTR-V30M-Cys) into NNTTR between 0 and 24 h after the removal of free cystine by SEC. The results show how the NNTTR fraction increases over time. The approach interestingly also provides a system to explore the effects of external thiol modulating agents. Figure 5, *B*–*E* demonstrates the impact of the different reducing agents GSH, NAC, BME, and TCEP on NNTTR formation. Figure 5*F* shows the effect on NNTTR formation as a function of cystine.

The results show that the reducing agents all efficiently suppressed the formation of NNTTR from rTTR-V30M-Cys and illustrate how either reducing the disulfides using *e.g.* TCEP or by saturating the cysteine using a stochiometric excess of thiols, suppresses the formation of NNTTR.

### Dilution of plasma strongly potentiates NNTTR formation by lowering the effect of molecular crowding

**Figure 6:**
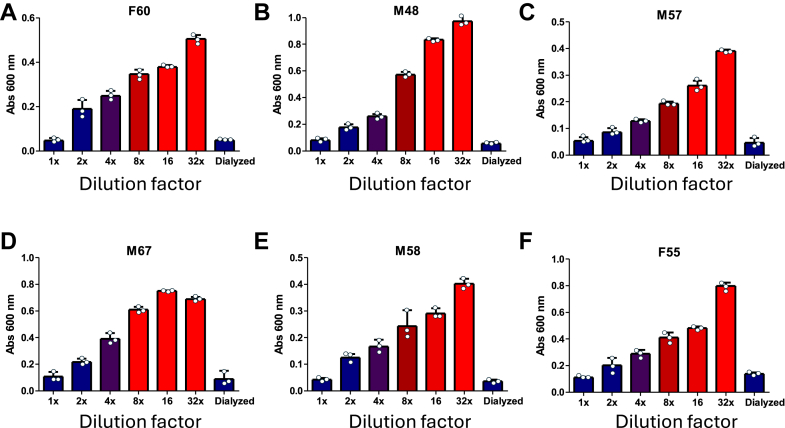
**Dilution of human plasma in PBS-EDTA strongly promotes NNTTR formation.** NNTTR formation was investigated using a sandwich-based ELISA with MAB_39-44_ on plasma from 6 *ttr-val30met* carriers *A*–*F*, shown as a function of 1X–32X dilution using PBS-EDTA, or dialyzed against PBS-EDTA using a 3.5 kDa membrane. All samples were incubated for 24 h at 37 °C and normalized to 5000X dilution using PBS-T before analysis. The sex (female (F) and male (M) and age of the individuals are given in each figure. All experiments have been repeated at least 3 times. Error bars indicate the standard deviation. NNTTR, nonnative variants of TTR; PBS-T, PBS supplied with 0.3% Tween-20.

In plasma, a large fraction of TTR is conjugated with thiol-adducts, which would be expected to promote NNTTR formation through disulfide shuffling as shown above. However, our results show that the rate of NNTTR formation in plasma is strongly suppressed. Interestingly, this suppression was lifted upon dilution in PBS-EDTA, and upon incubation the NNTTR levels frequently increased by more than 1000%. Figure 6 illustrates the effect of diluting plasma from six different *ttr-val30met* gene carriers, followed by a 24-h incubation at 37 °C and analysis using the MAB_39-44_ sandwich ELISA.

This unexpected result implicated the presence of a protective factor in the undiluted plasma, raising the question of whether macromolecular crowding, due to the high protein concentration in plasma, could stabilize the native state of TTR and suppress its conversion into NNTTR by limiting flexibility. To explore this, a parallel set of plasma samples from the corresponding donors was dialyzed against PBS-EDTA using a 3.5 kDa cutoff membrane, which removed potential small molecules while maintaining the high protein concentration. The results, shown in the last bars of Figure 6, *A*–*F*, indicate that even after dialysis, the high protein concentration continued to suppress NNTTR formation, supporting the hypothesis that macromolecular crowding has a stabilizing effect on the native state of TTR.

### Stabilizing the native state of TTR through increasing the macromolecular crowding or tetramer-stabilizing drugs suppresses NNTTR formation

**Figure 7:**
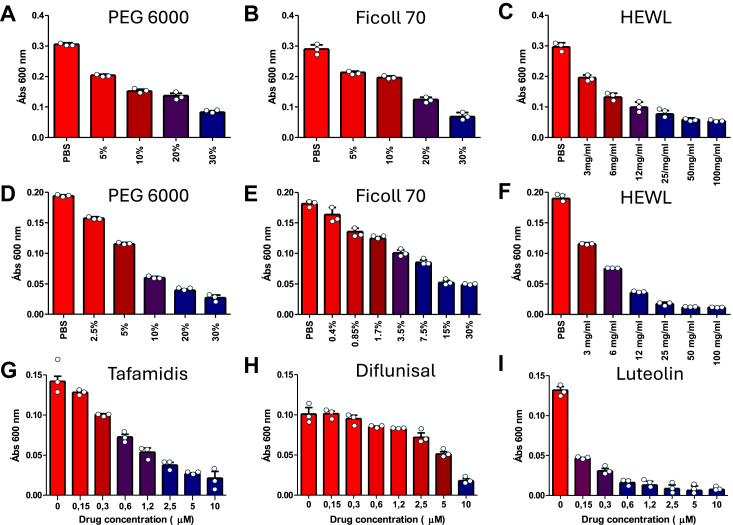
**The formation of NNTTR is effectively suppressed by macromolecular crowding and tetramer-stabilizing ligands.** Plasma from a *ttr-val30met* carrier was incubated for 24 h after 20X dilution in PBS-EDTA, pH 7.4 along with (*A*) PEG 6000 in concentrations of 0 to 30%, (*B*) Ficoll 70 in concentrations of 0 to 30%, and (*C*) HEWL in concentrations of 0 to 100 mg/ml. A corresponding analysis was performed on 4 μM of rTTR-V30M-Cys incubated along with (*D*) PEG 6000 in concentrations of 0 to 30% (*E*) Ficoll 70 in concentrations of 0 to 30%, and (*F*) HEWL in concentrations of 0 to 100 mg/ml. To probe the effect of tetramer stabilizing agents plasma from a *ttr-val30met* carrier was incubated for 24 h after 20X dilution in PBS-EDTA, pH 7.4 along with (*G*) tafamidis 0 to 10 μM. *H*, diflunisal 0 to 10 μM. *I*, luteolin 0 to 10 μM. All samples were diluted 5000X using PBS-T before analysis using a sandwich-based ELISA with MAB_39-44_. Error bars show standard deviations, and all experiments have been repeated at least 3 times. HEWL, hen egg-white lysozyme; PBS-T, PBS supplied with 0.3% Tween-20; NNTTR, nonnative variants of TTR; rTTR, recombinant TTR.

The lack of NNTTR formation in the dialyzed samples observed above suggests a suppression from molecular crowding in plasma. To further elucidate this, the well-known macromolecular crowding agents; PEG 6000, Ficoll 70, and hen egg-white lysozyme (HEWL), were evaluated in the same system. Figure 7, *A*–*C* shows the effect of 0 to 30% PEG 6000, 0 to 30% Ficoll 70, and 0 to 100 mg/ml HEWL on NNTTR formation in *ttr-val30met* plasma after diluting the plasma 20X. The results showed that all crowding agents induced a strong suppressing effect. The same result was also obtained for recombinant cysteinylated TTR; Figure 7, *D*–*F* shows the effect of 0 to 30% PEG 6000, 0 to 30% Ficoll 70, and 0 to 100 mg/ml HEWL on the formation of NNTTR from rTTR-V30M-Cys. Following the rationale that restrictions in the structural flexibility suppress the formation of NNTTR the same effect is expected from stabilizing ligands which are well-known to stabilize the native state of TTR. Figure 7, *H* and *I* shows the effect of the tetramer stabilizing drugs tafamidis, diflunisal, and luteolin on *ttr-val30met* plasma analyzed as above.

The acquired result strongly supports that stabilizing the native state of TTR prevents the structural flexibility required to bridge the distance between two thiols of the same tetramer and this stabilizing effect can be mediated by tetramer-stabilizing drugs, but interestingly also by the effect of macromolecular crowding.

### NAC effectively prevents the conversion of TTR into NNTTR in plasma

**Figure 8:**
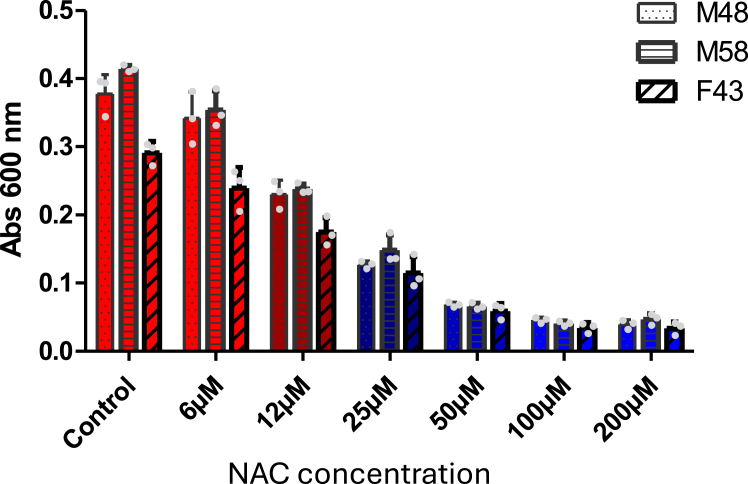
**Conversion of native TTR into NNTTR through dilution is effectively suppressed by NAC.** The propensity to form NNTTR was investigated in plasma from 3 *ttr-val30met* carriers, as a function of various concentrations of NAC. All samples were normalized to 5000X dilution using PBS-T before analysis for NNTTR using the MAB_39-44_ sandwich ELISA. All bars show standard deviations and all experiments have been repeated at least three times. NAC, N-acetylcysteine; NNTTR, nonnative variants of TTR; PBS-T, PBS supplied with 0.3% Tween-20; TTR, transthyretin.

From a therapeutic point of view, modulating the redox environment presents an interesting alternative or complement to current therapeutics. In this context, NAC is of specific interest since it is already an FDA-approved drug and food additive with a broad safety profile. Figure 8 illustrates the effect of NNTTR formation from three different *ttr-val30met* carriers after 20X dilution in PBS followed by incubation at 37 °C for 24 h in the presence of various concentrations of NAC. The response is suppressed by more than 50% already at 25 μM of NAC.

## Discussion

**Figure 9:**
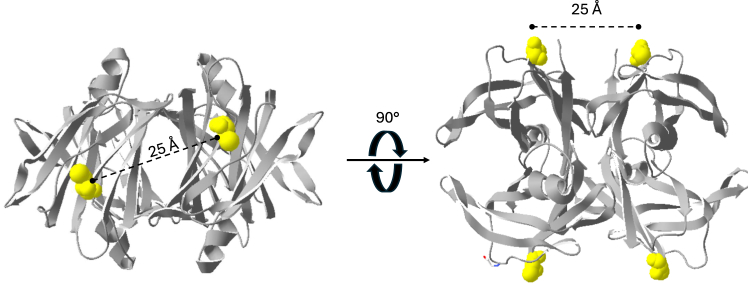
**Model of the native tetrameric assembly of TTR WT based on the crystal structure F141.pdb.** TTR is shown from two different views where the single cysteine located in position 10 on each subunit has been highlighted in *yellow*. The closest distance between two adjacent cysteines is around 25 Å. TTR, transthyretin.

The late onset of TTR-amyloidosis (1, 38) indicates that one or more additional factors control the misfolding and accumulation of TTR *in vivo*. This work has shown that the formation of the recently described pathogenic NNTTR species, discovered in the plasma of *ttr-val30met* carriers (19), depends on an intermolecular disulfide bond within the tetramer. A disulfide linkage is a characteristic feature of ATTR amyloid *in vivo* (21, 33) which recently also gained indirect structural support from cryo-EM analysis of *ex vivo* ATTR-V30M fibrils exposing a parallel β-sheet arrangement which as a consequence brings the thiol groups of adjacent polypeptides into proximity (39, 40). Although the fibrillar form of TTR can incorporate a disulfide bond within its structure, a disulfide bond between subunits within a native tetramer is not possible with a preserved native fold. A model of tetrameric TTR based on the crystal structure 1F41.pdb (41) is shown in Figure 9 where the cysteines have been highlighted in yellow. Based on the structure of TTR the closest distance between thiols is around 25 Å.

It is currently not possible to identify the specific pairing of cysteines within the tetramer although an intermolecular bridge between the closest pairs can be anticipated. The formation of an intermolecular disulfide bond between two subunits however irrespectively requires flexibility.

The ability of the disulfide-linked TTR to bind MAB_39-44_ reveals a structural change. Interestingly, the new fold also exposes a highly increased susceptibility to proteolytic digestion. Proteolytic fragmentation of TTR is a characteristic feature of ATTR-amyloid *in vivo* (21) but interestingly also a well-known promotor of TTR-fibril formation, both *in vitro* and *in vivo* (23, 25, 42). *In vivo*, the proteolytic digest predominantly targets the loop intervening strands C and D resulting in two dominating fragments. The specific protease responsible for this *in vivo* is yet to be determined although plasmin has been suggested as a potential candidate (23). Using trypsin, the digestion is not limited to the loop intervening strands C and D, but the results expose a vastly increased sensitivity to proteolytic digest where NNTTR is between 80 to 240 times more prone to degradation than the native fold.

Notably, the cysteine 10 of TTR has previously been assigned a critical role *in vivo* and by replacing it with a serine, amyloid formation was prevented in a transgenic mouse model for TTR amyloidosis (29).

The most tissue-damaging species within TTR-amyloidosis is not yet fully elucidated and while amyloid deposits can disturb organ function soluble oligomers have been shown to exert a cytotoxic effect (43, 44, 45). High levels of NNTTR were notably also associated with a worsened prognosis (19), indicating its role in the pathology.

As a consequence, NNTTR represents a highly attractive target for therapeutic intervention, both due to its potential role as a precursor to TTR amyloid *in vivo* and as an independent structure with intrinsic pathological properties.

This work reveals how native TTR can be converted into NNTTR, introducing two important new areas of investigation: the influence of the redox environment and factors affecting TTR's flexibility. In both areas, strategies for modulating these processes can interestingly be identified, pinpointing both potential risk factors as well as opportunities for therapeutic intervention.

From a redox chemistry perspective, the oxidation of two thiols into a disulfide bond involves the loss of two electrons, requiring the presence of an electron acceptor. Common electron acceptors *in vivo* include molecular oxygen, the oxidized form of GSSG, nicotine amide dinucleotide (NAD+), as well as reactive oxygen species (ROS) and reactive nitrogen species (RNS).

In plasma, a large proportion of oxidative adducts are already present on cysteine 10 of TTR, leaving only about 10 to 30% of TTR in a reduced state (26, 27, 28, 29, 30, 36, 37). The most common adducts are disulfide-linked and involve cysteinylation (40–50%), glutathionylation (10–20%), and cysteinglycinylation (5–10%). Although disulfides are strong covalent bonds, they are prone to disulfide shuffling where a deprotonated, thiolate, performs a nucleophilic attack on the disulfide. The result is a net exchange of the adduct, and since the pKa for most thiols is between 8 and 9 the reaction is pronounced also at neutral pH. Since the reaction with a free thiol in solution will compete with the corresponding reaction within a tetramer, the propensity to form NNTTR can simply be controlled by the concentration of free thiols in solution. We can show that by maintaining a stoichiometric excess of the thiols BME, NAC, or GSH, the formation of NNTTR can be strongly suppressed. An equally strong suppressing effect was also observed by the reducing agent TCEP which instead replaces the adduct with hydrogens and thus prevents disulfide-formation.

Although the conditions in plasma may appear optimal to form NNTTR, we can here expose that the conversion rate from native TTR into NNTTR is heavily suppressed through the effect of macromolecular crowding. The effect occurs in solutions where high concentrations of macromolecules restrict the excluded volume, *i.e.* the space occupied by proteins. A high number of nonspecific interactions thus prevent structural flexibility and consequently favor compact protein folds (46, 47). In the native structure of TTR, the thiol-groups of the cysteines located on different subunits are well separated in space (41), and the formation of a disulfide bond within a tetramer thus requires flexibility to bridge the distance. Human plasma has a protein concentration of around 60 to 80 g/liter, and we can here show that the crowded environment effectively suppresses the conversion of the intrinsic TTR having a native fold into NNTTR. However, through dilution into a noncrowded media, such as PBS, the effect is lowered and in several of the investigated plasmas, the resulting increase in NNTTR concentration is more than 1000%. The loss of stability through dilution can however easily be restored using other crowding agents such as PEG 6000, Ficoll 70, or HEWL, verifying the mechanism.

The higher propensity of TTR-V30M to convert into NNTTR as compared to TTR-WT in plasma can likely be explained by its lower stability. This is supported here by a comparison between rTTR-WT and rTTR-V30M regarding their propensity to convert into NNTTR as a function of diamide, shown in supporting information, Fig. S1.

A reduction of the flexibility can however also be accomplished by tetramer-stabilizing drugs, and by including either diflunisal, tafamidis, or luteolin, we show that NNTTR formation becomes effectively suppressed. This firmly verifies the hypothesis and exposes the clinical potential of tetramer stabilizing ligands also regarding the prevention of NNTTR.

Promoting NNTTR through dilution into a less crowded media may consequently contribute to the misfolding and aggregation of TTR. Human plasma is in equilibrium with the fluids of the interstitial space, *via* capillary exchange, which facilitates a regulated transport of both proteins and metabolites to the tissue. The protein concentration within the interstitial fluids is typically lower and only represents 30 to 40% as compared to plasma (48) and unless counterbalanced by other macromolecules or a more reducing environment NNTTR formation is enhanced. The lower total protein concentration may then potentially explain the formation of TTR-amyloid in the tissue. A similar explanation can be suggested regarding the vitreous body of the eye, which also is a characteristic site of TTR-amyloidosis (49) and where the overall protein concentration is low. A lack of molecular crowding can potentially also explain why ATTRv-amyloid may form in the brain (50, 51) since the total protein concentration of the cerebrospinal fluid only is around 0.5 mg/ml and thus only exerts a negligible stabilizing effect.

The ability of TTR to convert into amyloid correlates inversely with the stability of the molecule. Since adducts on cysteine 10 also may affect the stability of TTR this has previously been investigated as a potential risk factor regarding TTR-amyloidosis (36, 52). In this context, it is interesting to note that none of the adducts analyzed here, including NAC, BME, cysteine, and the comparatively large GSH, could facilitate a conformational change resulting in reactivity with MAB_39-44_. This suggests that all these adducts are comparatively well accommodated in the native conformation, compared to a disulfide linkage between two subunits of a tetramer.

The environment within the interstitial space, as well as within the vitreous body of the eye, is complex and greatly influenced by local factors. Macromolecules such as proteoglycans and hyaluronans are abundant and may also act as crowding agents. Interestingly, both hyaluronan and proteoglycans have previously been discovered in proximity to TTR-amyloid deposits *in vivo* (53). The proteoglycan heparan sulfate has even been suggested to accelerate TTR-amyloid formation in human kidneys (54); this may seem inconsistent with the findings presented herein, but the effect of macromolecular crowding frequently differs between folded and unfolded polypeptides. While folded proteins typically attain higher stability as a function of crowding (55, 56), disorganized and unfolded polypeptides are instead often promoted to aggregate by the same mechanism (57, 58, 59, 60). It has been demonstrated that denaturing conditions may cause the stabilizing effect of macromolecular crowding agents on the native fold to shift, and instead favor an aggregated state (56, 61). The interaction between NNTTR and various crowding agents will be the topic for future investigations, where also the combined effect of partial proteolytic cleavage will be investigated since it inevitably prevents a conversion back to its native state.

From the present study, we can conclude that the ability of TTR to form NNTTR is controlled by a delicate balance modulated by the redox environment, macromolecular crowding, and indirect also proteolysis. In humans, accumulation of amyloid is, however, frequently prevented until middle age or higher suggesting that the onset could potentially be attributed to changes in one or more of these parameters. In this context, changes in ROS and RNS are of special interest since they are well-known to increase with age. A large proportion of ROS, and thus indirectly also RNS, is produced by the electron transport chain during mitochondrial respiration. This results in the formation of superoxide anions, hydrogen peroxide, hydroxyl radicals, and (indirectly) peroxynitrite, and nitric oxide. Although ROS and RNS are required for several different cellular functions (62), their tightly regulated homeostasis is frequently disturbed as a function of age (63). Interestingly, previous studies found a correlation between mitochondrial haplogroups and the risk of developing TTR-amyloidosis (64, 65, 66). A possible explanation is that a genetic trait that favors a higher leakage of ROS, adversely impacts the redox state *in vivo*, thus increasing the conversion rate of TTR into NNTTR. *In vivo* ATTR deposits correlate with lipid peroxidation, seen as 4-hydroxy-trans-2-nonenal adducts (HNE) (67) and 8-hydroxy-2′deoxyguanosin (8-OhdG) (68) modifications, both of which are markers for the presence of ROS. Notably, a study that treated a transgenic mouse model for TTR-amyloidosis with carvedilol, a β-blocker with strong antioxidizing effects, found a reduction in both 4-hydroxy-trans-2-nonenal adducts and 8-hydroxy-2′deoxyguanosin adducts, as well as the total load of TTR deposits (69).

*In vivo*, ROS, and RNS are counteracted by enzymes such as catalase, thioredoxin, and superoxide dismutase (70), as well as GSH (71). Lower activity in these systems may thus also result in a more oxidative environment. Concerning the production of GSH as well as several antioxidating enzymes, cysteine is vital. The synthesis of cysteine is linked to the availability of vitamins B6, B9, and B12 (72), and deficiencies in any of these vitamins may thus also affect the ability to produce cysteine, and in particular vitamin B12 is frequently reduced with age (73).

Regarding therapeutic agents the FDA-approved drug and food additive NAC is of specific interest, both due to its efficacy to directly prevent the NNTTR formation, but also regarding its ability to promote production of GSH representing the dominating reducing agent *in vivo*. *In vivo*, NAC is rapidly converted to cysteine; it has a broad safety profile and can be administered at high doses (74). Interestingly, the oxidation of TTR in plasma has previously been investigated as a function of NAC in patients subjected to hemodialysis (75). A total amount corresponding to 5 g of NAC was administered intravenously during one dialysis session. The relative concentration of unmodified TTR increased, while the levels of cysteinylated and cysteinglycinylated TTR both decreased significantly (75). NAC may thus suppress NNTTR and potentially also TTR-amyloid formation *in vivo*. Although these results, in analogy to our findings on NNTTR, show that NAC can be used to reduce TTR, in plasma, the redox state is tightly controlled by the red blood cells (76). Future analysis of NAC, as a therapeutic approach to prevent TTR-amyloidosis, should therefore focus on its effect in the interstitial fluids rather than plasma. The slow progress of TTR-amyloid formation *in vivo* indicates that the equilibrium is delicate and that also a small adjustment of the imbalance between the formation and clearance of misfolded TTR can have a pronounced effect. NAC may therefore be a potential prophylactic avenue to postpone disease onset of symptoms. The effect of NAC is also not anticipated to interfere with current therapies, and combined treatments could therefore also be envisioned.

**Figure 10:**
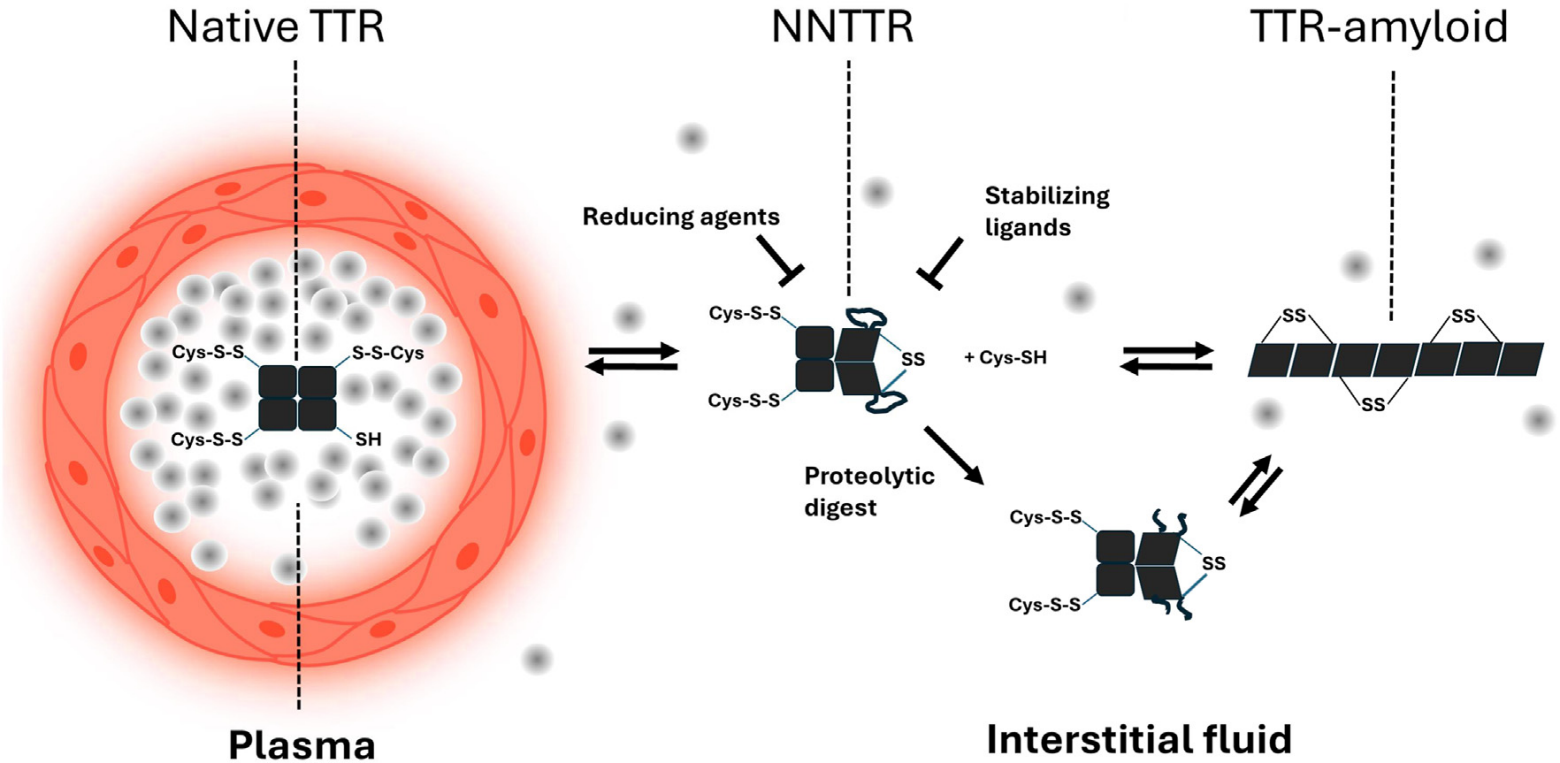
**A simplified schematic illustration showing how native TTR is stabilized by the highly crowded environment of human plasma.** The diffusion of TTR from the vascular system into the interstitial space lowers this stabilizing effect and increases the formation rate of NNTTR. The figure illustrates how the process can be suppressed by both reducing agents as well as stabilizing ligands. The novel epitopes exposed on NNTTR are susceptible to proteolytic digest and although both full-length TTR as well as fragments after proteolytic digest can form amyloid the latter cannot reverse to its native state. NNTTR, nonnative variants of TTR; TTR, transthyretin.

Taken together, this study demonstrates that the recently identified NNTTR, found in the plasma of *ttr-val30met* carriers, relies on an intermolecular disulfide bond within the tetramer. Its alternative fold exhibits high sensitivity to proteolysis which is a key feature of TTR-amyloid *in vivo* and a strong factor in promoting amyloid formation. We show that oxidizing agents can promote NNTTR while it can be effectively suppressed by reducing conditions. A disulfide bond within the tetramer is however incompatible with the native fold and the conversion thus requires flexibility. TTR-V30M has a lower stability and can more easily convert into NNTTR than TTR wt. Stabilizing the native state by the TTR-stabilizing ligands such as tafamidis, diflunisal, or luteolin effectively prevents the conversion into NNTTR. Interestingly, NNTTR conversion in plasma is also strongly suppressed by the effect of macromolecular crowding through the high protein concentration in plasma. Dilution of the plasma releases this suppression, linking amyloid deposits to areas such as the interstitial space and vitreous body of the eye, where overall protein concentration is lower. These findings propose a mechanistic route for TTR-amyloid formation *in vivo* and expose a novel target to suppress TTR misfolding, with both therapeutic and prophylactic implications. A schematic representation of the proposed pathway of TTR-amyloid formation, including the effect of redox, macromolecular crowding, and proteolytic digestion is presented in Figure 10.

## Experimental procedures

### Preparation of recombinant TTR

Recombinant TTR-WT and TTR-V30M (AlexoTech) were dissolved in 8 M of urea, containing 20 mM phosphate buffer and 150 mM NaCl, pH 7.4. The dissolved protein was subjected to SEC (Superose 6 10/300, Cytiva), equilibrated with 20 mM phosphate buffer and 150 mM NaCl (PBS), pH 7.4. Fractions of tetrameric TTR were collected. The molecular weight of the TTR was verified by mass spectroscopy (LC/MS TOF 6230B, Agilent).

### Detection of NNTTR using a sandwich-based ELISA with MAB_39-44_

The mouse monoclonal antibody MAB_39-44_ (AlexoTech) recognizes a cryptic epitope on TTR, located within the Asp39-Phe44 (32). Detection of both recombinant and plasma-derived NNTTR was performed through a sandwich-based ELISA, in a similar approach as previously described (32). In brief, MAB_39-44_ was coated on clear Nunc 96-well microtiter plates (Thermo Fisher Scientific) in PBS overnight at 4 °C, at a concentration corresponding to 4 μg/ml. Unbound sites were blocked using PBS supplied with 0.3% tween-20 (PBS-T) containing 2% dry milk (Semper), hereafter referred to as “blocking buffer”. The plates were subsequently washed three times with PBS-T. This washing procedure was repeated after all subsequent steps. Plasma samples and recombinant TTR were dissolved in PBS-T and incubated on the plates for 45 min. Identification of bound TTR was performed using the polyclonal rabbit anti-TTR_49-127_ antibody (AlexoTech), diluted 5000X in blocking buffer. Bound rabbit anti-TTR_49-127_ was identified using an anti-rabbit IgG HRP (Thermo Fisher Scientific), diluted 5000X in blocking buffer, incubated for 45 min, and the plate was washed as described above. A colorimetric approach based on 3,3′,5,5′-Tetramethylbenzidine (Agrisera) with absorbance at 600 nm was used to identify bound TTR.

### Collection of human plasma

The collection of human plasma was approved by the Swedish Ethical Review Authority (reference numbers 2022–04317–04 and 2022–06333–02). Plasma from *ttr-val30met* carriers and age-matched *ttr* controls was collected using EDTA tubes. All plasma samples were frozen at −80 °C within 1 hour of collection. The plasma samples were thawed only once before analysis. All human studies adhere to the Declaration of Helsinki principles.

### Immunoprecipitation using MAB_39-44_ and Western blot analysis

To isolate the NNTTR species in plasma from carriers of the *ttr-val30met* gene, as well as from recombinant TTR-V30M, 20 μg of the MAB_39-44_ antibody was dissolved in PBS-T and bound to 50 μl of protein-G coupled agarose (Cytiva). The beads were washed 10 times with 1 ml PBS-T using a 45 μm spin column. To isolate the MAB_39-44_-reactive species from plasma, 50 μl of plasma from five different *ttr-val30met* carriers was incubated with the prepared MAB_39-44_ protein-G agarose complex at room temperature for 15 min. The beads were subsequently washed 10 times with 1 ml of PBS-T, and bound material was eluted through the addition of 100 μl of 8 M of urea dissolved in 4% SDS, 20% glycerol, 0.1 M Tris, 0.1% bromophenol, pH 6.8, which also served as the loading buffer for the subsequent SDS-PAGE analysis. Where indicated, 2% BME was included, and all samples were heated to 98 °C for 10 min followed by separation using SDS-PAGE 4 to 20% gradient (Bio-Rad). After separation on SDS-PAGE, the proteins were transferred to a 0.2 μm polyvinylidene fluoride membrane (Bio-Rad) and analyzed using Western blotting according to standard procedures. In brief, after the transfer of the proteins, unbound sites on the membrane were blocked by incubation in the above-described blocking buffer and washed for 3∗5 min in PBS-T. The washing procedure was repeated after all the subsequent steps and bound TTR was identified using the polyclonal rabbit anti-TTR_49-127_ (AlexoTech), diluted 5000X in blocking buffer followed by incubation for 45 min. Detection was accomplished by an anti-rabbit IgG HRP-labeled antibody (Thermo Fisher Scientific) dissolved 5000X in blocking buffer and incubated for 45 min. Visualization of bound antibodies was performed using enhanced chemiluminescence (Agrisera) using a ChemiDoc reader, (Bio-Rad), according to standard procedures.

Immunoprecipitation and Western blot analysis of recombinant NNTTR were performed according to the same procedure as described above, using 50 μl of 5 μM recombinant TTR-V30M incubated overnight at 37 °C in PBS, supplemented by 40 μM of diamide (Sigma-Aldrich).

### SEC of native TTR and NNTTR

The preparation of a sample with a high proportion of NNTTR was accomplished by incubation of rTTR-V30M dissolved in PBS at a concentration of 2 mg/ml, in the presence of 6M of urea and 200 μM of diamide (Sigma-Aldrich) followed by incubation at 37 °C for 4 h. This resulted in a conversion of around 70% of total TTR into disulfide-linked TTR. Urea was subsequently removed through buffer exchange using a PD 10 column, (Cytiva) and the refolded protein was analyzed by SEC using a Superose-6 column. A fully reduced native rTTR-V30M incubated without diamide, but with 1% BME, was employed as a control. To illustrate the proportion of disulfide-linked TTR in the fractions from the NNTTR sample an SDS-PAGE analysis, performed as described above under nonreducing conditions, is included under the chromatogram.

### Proteolytic digest of TTR

rTTR-V30M dissolved in PBS at a tetrameric concentration corresponding to 30 μM was incubated with 200 μM diamide for 4 h at 37 °C. After incubation, the protein was subjected to SEC. Superose-6 10/300, (Cytiva) equilibrated in PBS, resulting in a single peak corresponding to the exclusion volume of a tetramer. The protein was subsequently diluted to 4 μM and aliquoted in seven tubes in the presence of bovine trypsin, (Sigma-Aldrich), as indicated in the figure, followed by incubation at 37 °C for 1 h. Proteins were separated on SDS-PAGE and stained with Coomassie brilliant blue 250R (Sigma-Aldrich) according to standard procedures.

### Purification of disulfide-linked dimers of TTR

Subsequently, 5 mg of recombinant TTR-V30M was incubated overnight in 5X stoichiometric excess of diamide and dissolved in 8 M of urea to acquire free monomers and disulfide-linked dimers in solution. The dissolved TTR was then separated using a 5/5 Mono-Q column (Cytiva). The equilibration and elution buffer both contained 10 mM Tris and 6 M of urea, pH 8.0, while the elution buffer also contained 1 M of NaCl. Using a 10-column volume gradient between 0 and 1 M NaCl, the monomeric and disulfide-linked TTR were separated. The dimeric fraction was subsequently subjected to a buffer exchange using a PD 10 column (Cytiva) equilibrated with PBS, pH 7.4.

### Atomic force microscopy analysis of recombinant TTR

To examine the ability of disulfide-linked TTR dimers to convert into amyloid, the isolated disulfide-linked fraction of TTR-V30M was incubated in PBS at a protein concentration corresponding to 0.3 mg/ml for 72 h at 55 °C. The morphology of the acquired assemblies was analyzed using AFM. Samples were applied onto a freshly cleaved mica surface, incubated for 2 min, and washed four times with deionized water to remove buffer and salt. The surface was subsequently analyzed using a Nanowizard 4 XP BioScience AFM (Bruker), equipped with a motorized sample stage and top-view optical module.

### Conjugating cysteine to position 10 on TTR

Recombinant TTR-V30M was dissolved and purified using SEC, as described above. A solution corresponding to 20 μM of tetrameric recombinant TTR-V30M was mixed with a saturated suspension of cystine (Sigma-Aldrich), buffered to pH 7.5 using 0.5 M Na_2_HPO_4_. The protein was incubated in the suspension of cystine at room temperature for 3 h, followed by removal of free cystine using a Superose-6, 10/300 column (Cytiva) and resulted in around 90% cysteinylation based on mass-spectroscopy analysis (LC/MS TOF 6230B, Agilent).

### Probing the effect of crowding on NNTTR formation

Plasma from *ttr-val30met* carriers was dissolved and diluted 20X in PBS-EDTA, pH 7.4 in the presence of 0 to 30% polyethylene glycol 6000 (Sigma-Aldrich), 0 to 30% Ficoll 70, (Sigma-Aldrich) or 0 to 100 mg/ml HEWL (Sigma-Aldrich).

All plasma samples were incubated for 24 h, followed by analysis using the sandwich-based ELISA with MAB_39-44_. Recombinant TTR-V30M conjugated with cysteine was incubated as above, at a tetrameric concentration corresponding to 4 μM, followed by analysis using the sandwich-based ELISA with MAB_39-44_.

### Investigating the effect of tetramer-stabilizing drugs on NNTTR formation in plasma

Plasma from a *ttr-val30met* carrier was diluted 20X in PBS-EDTA, pH 7.4 containing various concentrations of the tetramer-stabilizing drugs, diflunisal, tafamidis, and luteolin. All samples were incubated at 37 °C for 24 h, followed by analysis using the sandwich-based MAB_39-44_ ELISA.

## Data availability

All data are included in the manuscript and supporting information.

## Supporting information

This article contains supporting information.

## Conflict of interest

S. W. J., I. A., and A. O. are the owners of a patent application based on the described findings. PCT/SE2024/050271. The other authors declare that they have no conflicts of interest with the contents of this article.
