# Supplementary material for: Misfolding of transthyretin in vivo is controlled by the redox environment and macromolecular crowding
Source: J Biol Chem. 2024 Nov 28;301(1):108031. doi: 10.1016/j.jbc.2024.108031 (PMC11732491; doi:10.1016/j.jbc.2024.108031)
Supplement: Supporting information [file mmc1.docx]

**Supporting information**


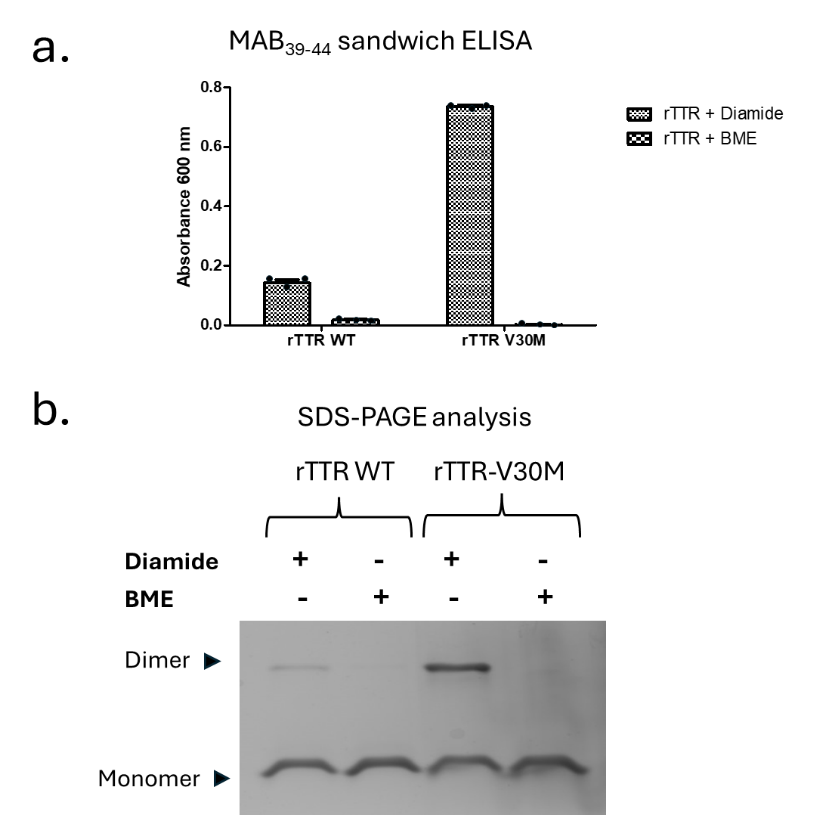


**Figure S1. Comparing the propensity of rTTR WT and TTR-V30M to convert into NNTTR as a function of diamide. (a)** Probing the relative levels of NNTTR formed after incubation of 4µM of rTTR-WT and rTTR-V30M in PBS, after 2 hours at 37°C in the presence or absence of 40 µM diamide or 1 mM BME, using the MAB39-44 sandwich based ELISA. Samples was diluted 4000X in PBS-T before ELISA. **(b)** SDS-PAGE of the above samples.
